# A Trojan horse nanoplatform overcoming delivery barriers for targeted gene immunotherapy of colorectal cancer

**DOI:** 10.3389/fbioe.2026.1947125

**Published:** 2026-09-24

**Authors:** Yao Wang, Yongjie Yu, Qingyan Deng, Dengyun Liu, Xiaofei Liang, Haitao Wang, Xinhao Zhou, Jun Ouyang, Yueming Huang, Lijun Xiang, Zhifen Guan, Bohao Cheng, Zhigang Guo, Zhifeng Yang, Naisheng Deng, Qixing Zhong, Guiyuan Xie, Kai Li, Zejun Wang, Weidong Li, Na Song

**Affiliations:** 1 Department of Gastrointestinal Surgery, Zhongshan City People’s Hospital, Zhongshan, China; 2 School of Materials Engineering, Changzhou Vocational Institute of Industry Technology, Changzhou, China; 3 Guangzhou Mercury Biomedical Technology Co., Ltd, Guangzhou, China; 4 Affiliated Hospital of Guizhou Medical University, Guiyang, China; 5 Shanghai University, Shanghai, China

**Keywords:** biomimetic nanoplatform, colorectal cancer, CRISPR/Cas9, PD-L1, SOX9

## Abstract

Programmed cell death ligand 1 (PD-L1) antibody has been shown to hold tremendous promise in curing metastatic colorectal cancer (CRC). However, the delivery efficacy of the targeted biologics limits this immunotherapy in a subset of CRC. Here we report a novel biomimetic ‘Trojan horse’ nano-delivery system for targeted gene-immunotherapy of colorectal cancer. The system comprises a core of gold nanoparticles and cationic liposomes encapsulating CRISPR/Cas9–sgSOX9, camouflaged with homologous CRC cell membranes for immune evasion and tumor tropism, and functionalized with PD-L1 antibodies to enhance tumor recognition and relieve immune suppression. *In vitro*, the nano-system potently suppressed CRC cell proliferation, migration and invasion. *in vivo*, it exhibited robust tumor-targeting specificity, favorable preliminary biosafety profile and marked inhibition of tumor growth in subcutaneous xenograft models. Mechanistically, the system remodeled the tumor microenvironment by promoting CD8^+^ T-cell infiltration and elevating effector cytokines IFN-γ and TNF-α. By seamlessly coupling precise gene delivery with potent immune activation, this nanoplatform offers a compelling therapeutic strategy for colorectal cancer therapy.

## Introduction

Colorectal cancer (CRC) is the third most commonly diagnosed malignancy and the second leading cause of cancer-related mortality worldwide, with metastatic disease responsible for over 90% of deaths (Heinlein et al., 2026; Wang et al., 2026). Although immune checkpoint blockade targeting the programmed cell death ligand 1 (PD-L1) pathway has revolutionized treatment for a subset of metastatic CRC patients (Ribas and Wolchok, 2018), particularly those with microsatellite instable or mismatched tumors, the vast majority of cases exhibit primary resistance (André et al., 2024; Yang and Chen, 2025; Lind et al., 2020). This limitation stems from the inherently immunosuppressive tumor microenvironment in CRC (Kennel and Greten, 2025), characterized by sparse T-cell infiltration, low tumor mutational burden and abundant myeloid-derived suppressor cells that collectively establish a cold tumor phenotype refractory to PD-L1 antibody monotherapy (Wu et al., 2024). Consequently, strategies capable of converting these immunologically cold lesions into hot, T-cell-inflamed tumors represent a critical unmet need in CRC therapeutics.

Transcriptional reprogramming of the tumor microenvironment through targeted disruption of master regulatory oncogenes represents a transformative strategy to convert immunologically cold colorectal tumors into hot (Yang J. et al., 2025). The SRY-Box Transcription Factor 9 (SOX9) has emerged as a pivotal dual function driver that not only sustains cancer stem cell self-renewal and epithelial mesenchymal transition but also actively sculpts an immunosuppressive niche, among the constellation of transcription factors governing colorectal cancer pathogenesis (Avendaño-Felix et al., 2023). SOX9 is markedly upregulated in colorectal carcinoma and its elevated expression correlates with aggressive clinicopathological features, metastatic dissemination, and poor patient prognosis. Mechanistically, SOX9 orchestrates immune evasion through multifaceted paracrine crosstalk. SOX9 promotes polarization of tumor-associated macrophages toward the M2 immunosuppressive phenotype via activation of the leukemia inhibitory factor receptor (LIFR) signaling axis and concomitantly suppresses CD8^+^ cytotoxic T lymphocyte infiltration while recruiting myeloid-derived suppressor cells (Zhong et al., 2026), thereby establishing a formidable physical and functional barrier to PD-L1 antibody efficacy. Furthermore, SOX9 maintains *Wnt*/*β*-catenin driven cancer stemness (Dai et al., 2020), which perpetuates a self-renewing reservoir of tumor-initiating cells capable of reconstituting the immunosuppressive microenvironment following therapeutic insult. Consequently, genetic ablation of SOX9 offers a rational approach to simultaneously dismantle this immunosuppressive fortress, eradicate cancer stem cells, and sensitize resistant tumors to immunotherapy (Chen et al., 2024). However, the clinical translation of SOX9-directed CRISPR–Cas9 gene editing has been severely constrained by the absence of safe and efficient delivery modalities capable of navigating the formidable biological barriers that plague systemic nucleic acid administration (Du et al., 2023; Aslan et al., 2024; Glass et al., 2017). Naked CRISPR ribonucleoprotein complexes suffer from rapid renal clearance, susceptibility to serum nuclease degradation, limited cellular uptake across the endothelial–stromal barrier, and substantial off-target editing in hepatocytes and other reticuloendothelial organs, necessitating the development of sophisticated vector systems that can encapsulate, protect, and precisely direct these macromolecular payloads to the tumor bed (Sharma et al., 2022). In this context, biomimetic ‘Trojan horse’ nanoplatforms, particularly those built upon gold nanoparticle–liposome hybrid cores and camouflaged with homologous tumor cell membranes (Peng et al., 2015), have garnered substantial interest as they exploit endogenous immune-evasion mechanisms, CD47-mediated ‘do not eat me’ signaling (Liu et al., 2019), and homologous tumor-homing capabilities to achieve selective payload delivery while minimizing premature immune recognition and reticuloendothelial system sequestration (Ju et al., 2025). The gold nanoparticle core provides a robust scaffold for CRISPR ribonucleoprotein condensation and potential photothermal sensitization (Gottimukkala et al., 2026; Cunningham et al., 2026), whereas cationic liposomal encapsulation ensures protection against nuclease attack and facilitates endosomal escape following cellular internalization. Concurrently, surface functionalization with PD-L1 antibodies presents an elegant dual-mechanism strategy: it not only directs active targeting to PD-L1-expressing tumor epithelial cells and tumor-associated myeloid populations but also concurrently relieves local immune suppression by blocking the PD-1/PD-L1 inhibitory axis, thereby amplifying CD8^+^ cytotoxic T-cell effector function and promoting promising immunomodulatory effects in the tumor microenvironment (Sun et al., 2018; Wang et al., 2024a; Li et al., 2015). Recent reviews have highlighted the potential of nanomaterial-based approaches for modulating PD-L1 expression in the tumor microenvironment, further supporting the rationale for integrating PD-L1 targeting into nanoplatform design (Inam et al., 2025). Although discrete advances have been realized in membrane-coated nanoparticles for chemotherapeutic delivery and in antibody-conjugated liposomes for immune modulation, the seamless integration of precise SOX9 gene editing with PD-L1-targeted immunotherapy within a single, unified biomimetic nanoplatform remains largely unexplored (Wang et al., 2025), particularly in the context of colorectal cancer models that faithfully recapitulate the anatomical constraints, immunosuppressive tumor microenvironment, and metastatic progression characteristic of human colorectal malignancy, creating an urgent need for integrated delivery solutions that can simultaneously edit oncogenic drivers and unleash adaptive anti-tumor immunity.

Here, we report a biomimetic ‘Trojan horse’ nano-delivery system (AuCS-PD) that couples CRISPR/Cas9 mediated SOX9 gene editing with PD-L1 targeted immunotherapy for CRC (Figure 1). The system features a core of gold nanoparticles and cationic liposomes encapsulating CRISPR/Cas9 sgSOX9, camouflaged with homologous CRC cell membranes to achieve immune evasion and tumor tropism, and functionalized with PD-L1 antibodies to enhance tumor recognition and relieve immune suppression. We demonstrate that this platform potently suppresses CRC cell proliferation, migration and invasion *in vitro*, exhibits robust tumor-targeting specificity, favorable preliminary biosafety profile and marked inhibition of tumor growth in subcutaneous xenograft models *in vivo*, and remodels the tumor microenvironment by promoting CD8^+^T-cell infiltration and elevating effector cytokines IFN-γand TNF-α.By ingeniously integrating precise gene delivery with immune modulation, this strategy offers a compelling therapeutic paradigm for CRC.

**FIGURE 1:**
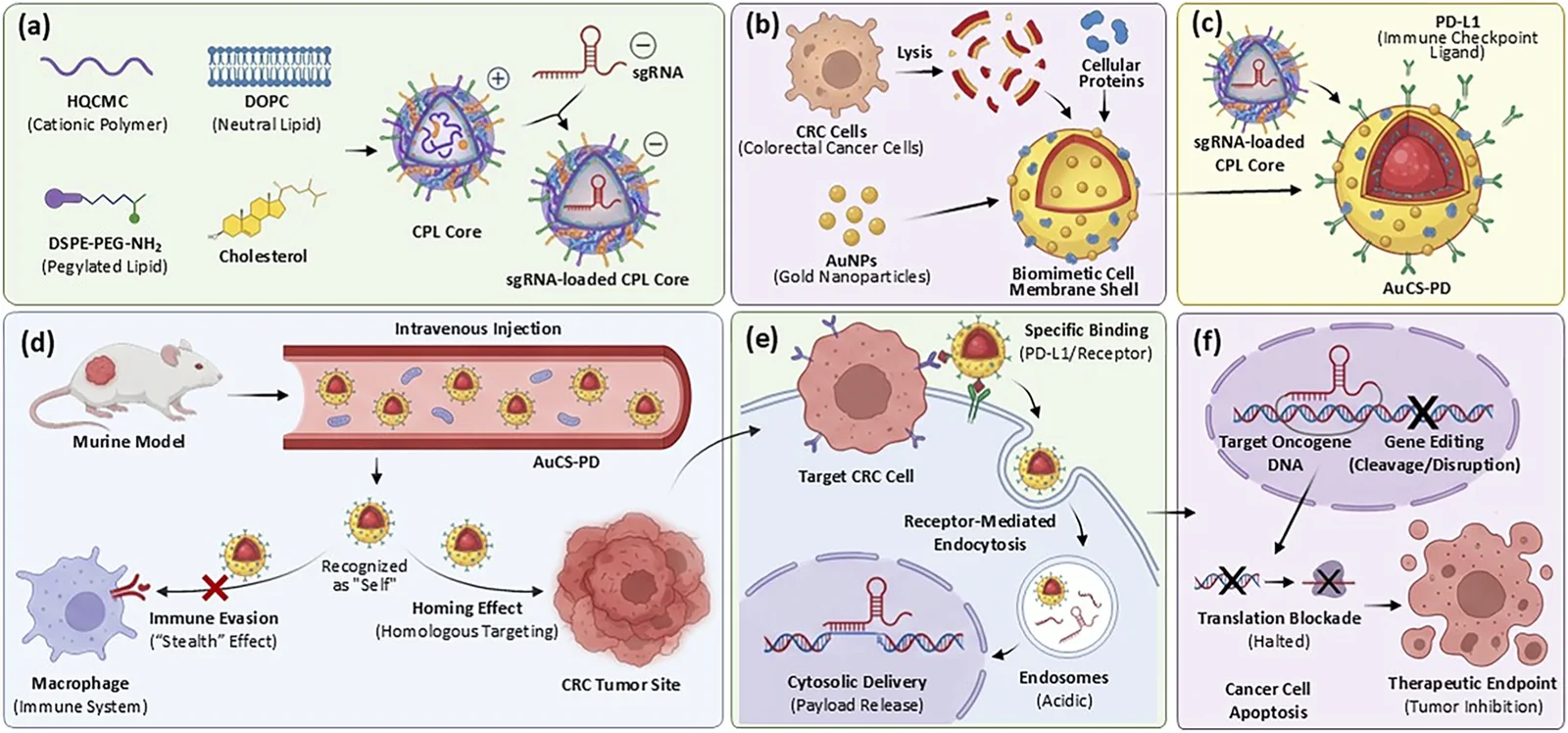
A novel nanocomposite system for curing metastatic colorectal cancer. **(a)** construction of the sgRNA-loaded polymer-lipid core: the four components, i.e. HQCMC (an amino-modified cationic polymer), DOPC (a neutral phospholipid), DSPE-PEG-NH_2_ (an amino-functionalized PEG lipid for stabilization and bioconjugation), and Cholesterol, undergo self-assembly in solution, driven by hydrophobic and electrostatic interactions, to form a positively charged polymer-lipid nanocore (CPL core), the negatively charged sgRNA (a critical negatively charged component of the CRISPR gene-editing system) is encapsulated into or strongly adsorbed onto the positively charged CPL core (sgRNA-loaded CPL core); **(b)** construction of the biomimetic cell membrane shell: CRC cells (colorectal cancer cells) are lysed to extract their natural cell membrane along with specific cellular proteins. Gold nanoparticles (AuNPs) are then integrated with these extracted cellular components to formulate a biomimetic membrane shell embedded with AuNPs and natural tumor antigens; **(c)** construction of the integrated Au/PD-NPs: the sgRNA-loaded CPL core is coated with the prepared biomimetic CRC cell membrane, the outermost surface of the nanoparticle is further functionalized with PD-L1 (an immune checkpoint ligand, utilized here for specific targeting or immune modulation). **(d)** immune escape and homing effect: Immune escape, following intravenous injection, the biomimetic coating of autologous CRC cell membranes acts as an immunological cloak, the host’s immune system (such as macrophages of the reticuloendothelial system) recognizes the nanoparticles as “self,” thereby preventing phagocytic clearance and significantly prolonging systemic circulation time. Homing effect, Leveraging the homotypic adhesion properties of cancer cell membranes, the disguised nanoparticles act as guided missiles, autonomously targeting and highly accumulating at the solid CRC tumor site. **(e)** cellular uptake and cytosolic delivery: Upon reaching the tumor microenvironment, the surface modifications (e.g., PD-L1) and natural membrane proteins facilitate specific binding to homologous receptors on the target CRC cells. The nanoparticles are internalized by the cancer cells via receptor-mediated endocytosis. Once inside the intracellular vesicles (e.g., acidic endosomes), the nanoparticles disassemble, successfully achieving cytosolic delivery by releasing the gene-editing payload (sgRNA) into the cytoplasm. **(f)** Gene editing and translation blockade: The released sgRNA precisely navigates to the specific target DNA sequence of a critical oncogene or survival gene within the nucleus.

## Results

### Construction and characterizations of AuCS-PD

In this study, the biomimetic nanocomposite (AuCS-PD) integrates gold (Au) nano-cationic lipid CRISPR complexes with CRC cell membrane encapsulation and PD-L1 antibody conjugation, enabling dual-targeted SOX9 ablation to effectively reprogram the colorectal cancer (CRC) immune landscape. The TEM micrograph revealed a particle size of approximately 100 nm under dry conditions (Figure 2a). In aqueous solution, DLS measurements gave a larger hydrodynamic diameter of 169.7 ± 3.4 nm with a polydispersity index (PDI) of 0.17 ± 0.02 (n = 3) (Figure 2d), reflecting the hydrated lipid-membrane shell and solvation layer surrounding the gold core, and 13.3 ± 3.2 mV surface potential (Figure 2e). The nucleic acid-binding capacity of AuCS-PD was characterized via a gel shift assay. As the weight ratio increased, the mobility of sgRNA was progressively inhibited, reaching complete retardation at ratios above 5 (Figure 2f). When the AuCS-PD concentration increased from 5 μg/ml to 200 μg/ml (Figure 2g), the hemolytic activity increased from 1.11% to 4.42% (Figure 2h), and the hemolytic activity of all experimental concentrations and different groups was lower than 6%, indicating that the prepared AuCS-PD has high biosafety and good biocompatibility. The zeta potential of 13.3 ± 3.2 mV, together with the low hemolytic activity (<5%, Figure 2h) and prolonged *in vivo* circulation (Figure 6B), collectively supports the colloidal stability of AuCS-PD under physiological conditions.

**FIGURE 2:**
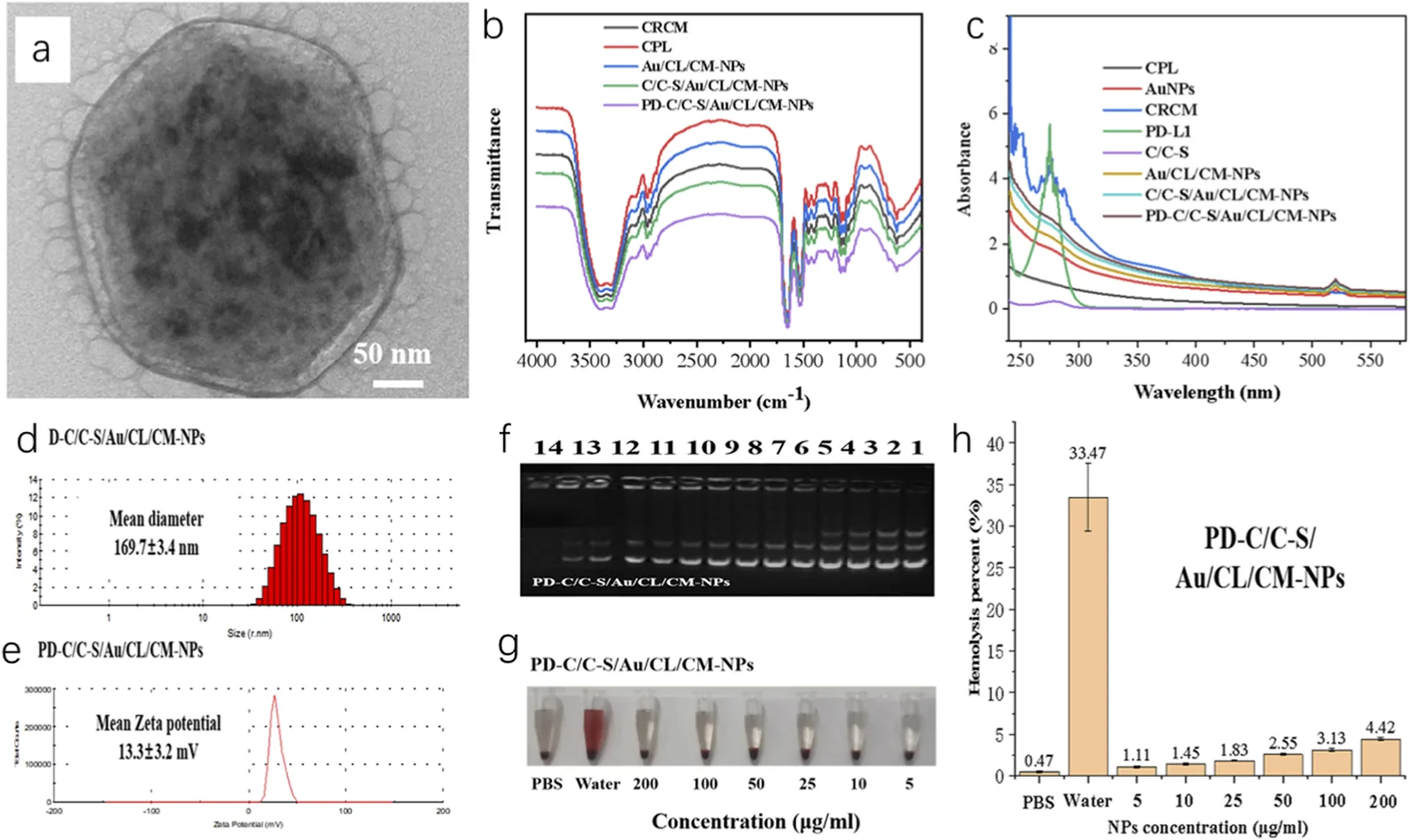
Characterization of AuCS-PD nanocomposite system. **(a)** TEM micrograph of AuCS-PD nanoparticles (50 μm). **(b)** Fourier transform infrared spectra of AuCS-PD. **(c)** UV-vis absorbance of AuCS-PD. **(d)** Size distributions of AuCS-PD (PDI = 0.17 ± 0.02, n = 3). **(e)** Zeta potential distribution of AuCS-PD. f, Gel electrophoresis analysis of different AuCS-PD: sgRNA weight ratios ranging from 1:1, 2:1, 3:1, 4:1, 5:1, 6:1, 7:1, 8:1, 9:1, 10:1, 11:1, 12:1, 13:1, 14:1. For particle size and zeta potential measurements (panels **(d,e)**), data were collected from three independent batches (n = 3 per group). Data are presented as mean ± SD. Statistical comparisons were performed using one-way ANOVA with Tukey’s post-hoc test; P < 0.001, P < 0.01, P < 0.05, ns = not significant (P > 0.05). **(f)** Gel electrophoresis analysis of different AuCS-PD:sgRNA weight ratios; **(g)** Photographs of hemolysis assay at different concentrations; **(h)** Quantification of hemolysis percentage.

### Cancer cellular uptake behavior

The AuCS-PD treatments on the PD-L1 overexpressing LOVO cells have higher FITC fluorescence intensity compared to the AuCS treatments, while the FITC intensity of the SW480 cells has negligible differences between the AuCS-PD and AuCS nanoparticles (Supplementary Figure S2). To demonstrate that high-affinity surface binding followed by receptor-mediated recognition and endocytosis enables the targeted delivery of various types of cargo, we loaded FITC (Figure 3) and cy3-labelled siRNA into PD-L1-nanocomposite, which were then added to SW480 and LOVO cells for 3 h. HCFM and quantitative analyses revealed that the delivery of the encapsulated cargo to the SW480 cells using AuCS-PD was achieved via the following steps. The multivalent binding of PD-L1 antibody to SW480 surface receptors-initiated receptor-mediated recognition, an internalization pathway that facilitates the entry of NPs into cells. The cellular increase in FITC fluorescence and the red fluorescence signals of the cy3-siRNA and FITC indicated that the cargo-loaded AuCS-PD were rapidly endocytosed by the SW480 cells and that PD-L1 promoted the efficient uptake of the NPs during the first 3 h. The cells that were treated with AuCS-PD accumulated larger amounts of siRNA and FITC in their nuclei compared with the cells that were treated with non-targeted NPs, possibly because the PD-L1-mediated recognition and internalization pathway. The preferential uptake of membrane-coated nanoparticles by homologous CRC cells further confirms that functional homotypic adhesion proteins are successfully preserved on the nanoparticle membrane coating.

**FIGURE 3:**
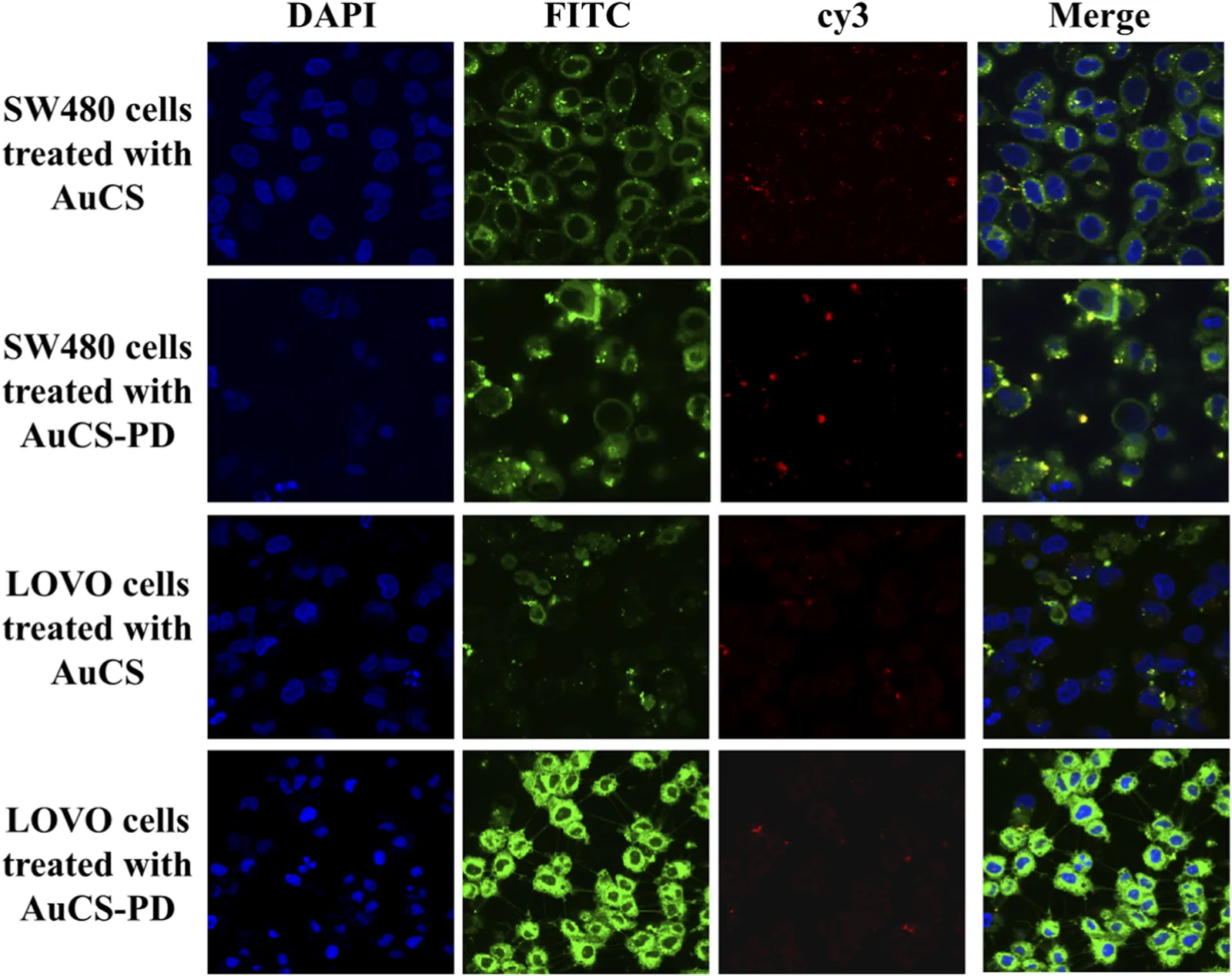
Localization and distribution of AuCS and AuCS-PD in SW480 and LOVO cells. Cells were cultured in a medium that contained FITC-labelled AuCS and AuCS-PD NPs in a glass bottomed dish (35 mm dish with a 14 mm bottom well) for 3 h, followed by treatment with DAPI dye for 5–10 min and subsequent examination by confocal microscopy. Quantification of fluorescence intensity was performed on at least 50 cells per group from three independent experiments (n = 3). Data are presented as mean ± SD. Statistical comparisons were performed using one-way ANOVA with Tukey’s post-hoc test; P < 0.001, P < 0.01, P < 0.05, ns = not significant (P > 0.05). Scale bars = 10 μm.

### Characterization of PD-Au/CL/CM-NPs as gene delivery vectors

The SW480 and LOVO lines were chosen to study the gene transfection efficiency of PD-Au/CL/CM-NPs (at the NPs/sgRNA weight ratios of 6/1). As shown in Figure 4, PD-L1 modified Au/CL/CM-NPs displayed higher FITC green fluorescence both in SW480 and LOVO cells compared with the PD-L1-free Au/CM/CPL-NPs. This implied that the enhanced fluorescence expression of PD-Au/CL/CM-NPs by SW480 and LOVO cells was due to the PD-L1 mediated endocytosis. In order to quantitatively test the gene transfection efficiency of PD-L1 modified Au/CL/CM-NPs and PD-L1-free Au/CM/CPL-NPs, luciferase expression experiments were also carried out (at NPs/DNA weight ratios of 6/1). The fluorescence expression mediated by Au/CM/CPL-NPs/FITC-sgRNA and PD-Au/CM/CPL-NPs/FITC-sgRNA complexes were influenced by cell lines. In 2 cell lines, the FITC expression mediated by AuCS-PD is 2–5 times higher than that mediated by Au/CM/CPL-NPs. These results suggested that AuCS-PD were able to selectively deliver foreign genes to cell over-expressing PD-L1.

**FIGURE 4:**
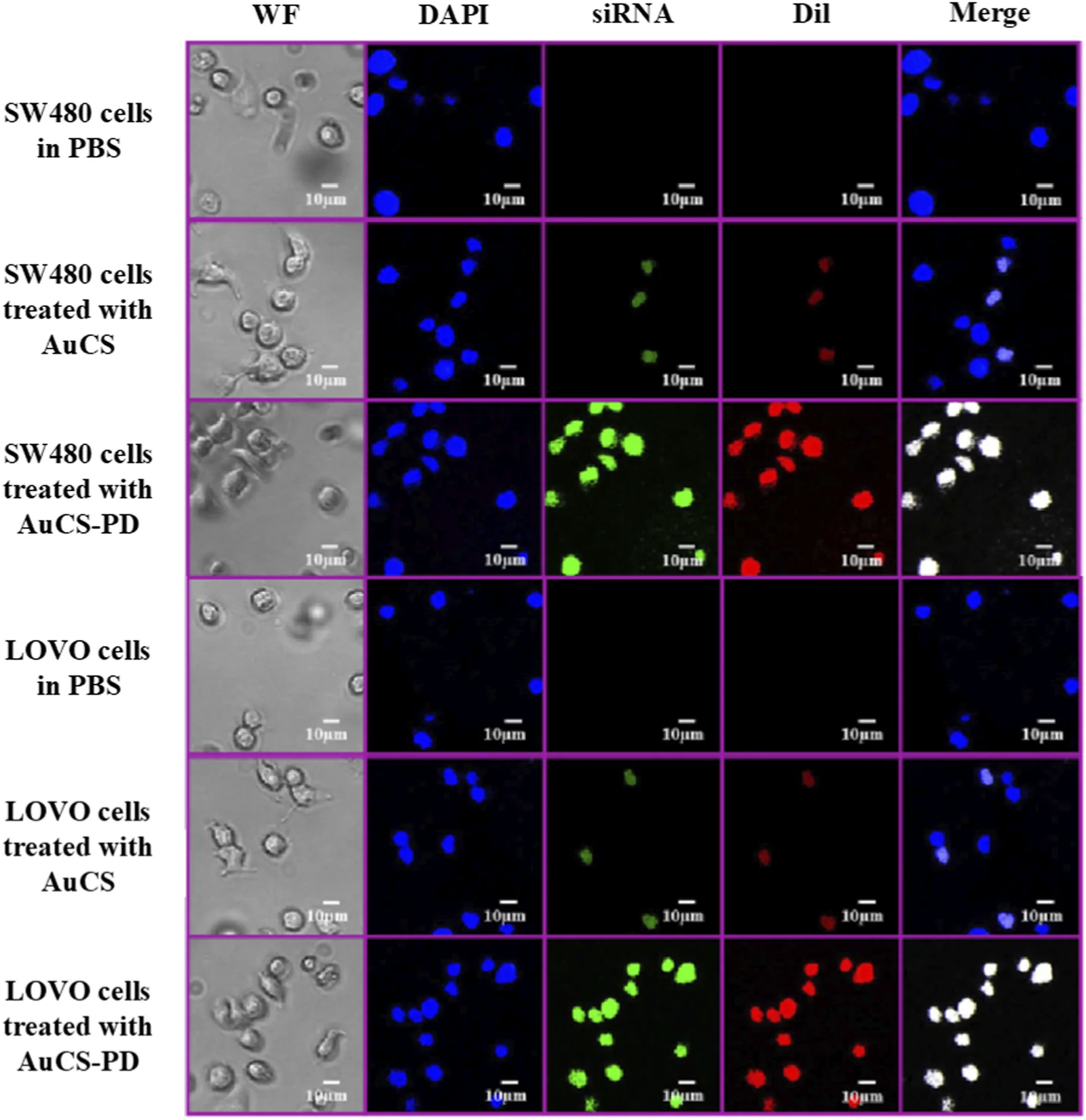
Fluorescence microscope images of SW480 and LOVO colorectal cancer cells treated with different nanoparticles for 30 min. Images are representative of three independent experiments (n = 3). Fluorescence intensity was quantified using ImageJ software. Data are presented as mean ± SD. Statistical comparisons were performed using one-way ANOVA with Tukey’s post-hoc test; P < 0.001, P < 0.01, P < 0.05, ns = not significant (P > 0.05). Scale bars = 50 μm.

### Inhibition effects on colorectal cancer cells growth *in vitro*

We found that AuCS and AuCS-PD nanoparticles did not significantly inhibit SW480 and LOVO cells growth *in vitro* at 10/50 μg/mL compared with the treatments of PBS and SOX9 (*P* > 0.05), whereas significant inhibition was observed at 100 and 200 μg/mL (*P* < 0.05) (Figures 5A,B). To further dissect the contribution of individual functional components to the antitumor activity of AuCS-PD, we evaluated the cell viability of LOVO cells treated with a series of intermediate nanoparticle formulations at a fixed concentration of 100 μg/mL (equivalent to 5 μg/mL sgRNA) for 48 h (Supplementary Figure S4; Supplementary Table S2). The carrier control without sgRNA or PD-L1 (Au/CL/CM-NPs) showed no significant cytotoxicity compared with the PBS control group (95.81% ± 9.89% vs. 101.35% ± 4.04%, P > 0.05), confirming the excellent biocompatibility of the nanocarrier itself. In contrast, the formulation containing sgRNA with cationic liposomes but without Au or membrane coating (C/C-S/CL-NPs) reduced cell viability to 57.45% ± 32.26% (P < 0.01), indicating that liposome-mediated sgRNA delivery contributes substantially to the antitumor effect. The addition of the gold nanoparticle core (C/C-S/Au/CL-NPs) yielded comparable cytotoxicity (58.55% ± 31.77%, P < 0.01), suggesting that the Au core primarily functions as a structural scaffold rather than directly enhancing gene delivery efficiency in the absence of photothermal stimulation. Notably, cancer cell membrane coating (C/C-S/Au/CL/CM-NPs) resulted in further enhanced cytotoxicity (52.20% ± 32.92%, P < 0.001), likely due to improved cellular uptake via homologous targeting (Zheng et al., 2025). The complete formulation with PD-L1 antibody functionalization (PD-C/C-S/Au/CL/CM-NPs, AuCS-PD) exhibited the strongest inhibition (44.10% ± 38.81%, P < 0.001), confirming that PD-L1-mediated active targeting synergizes with membrane-based homologous recognition to maximize the delivery of the CRISPR/Cas9-sgSOX9 payload (Wang et al., 2024b; Li et al., 2025). These hierarchical cytotoxicity data demonstrate that the therapeutic efficacy of AuCS-PD arises from the cooperative action of its multiple functional components, with the membrane coating and PD-L1 functionalization providing incremental but significant contributions to the overall antitumor activity. The annexin-V/PI double-staining assay results showed that the inhibition effects of AuCS and AuCS-PD nanoparticles on SW480 and LOVO cells growth was associated with cell apoptosis. The induction of apoptosis by the AuCS-PD nanoparticles was stronger than that by the AuCS nanoparticles. To dissect the individual contributions of each functional component to the overall antitumor activity of AuCS-PD, we performed a hierarchical control experiment using a series of intermediate nanoparticle formulations at a fixed concentration of 100 μg/mL (equivalent to 5 μg/mL sgRNA) for 48 h (Supplementary Figure S4). The carrier control without sgRNA or PD-L1 (Au/CL/CM-NPs, Group B) showed no significant cytotoxicity compared with the PBS control group (95.81% ± 9.89% vs. 101.35% ± 4.04%, P > 0.05), confirming that the nanocarrier itself is biocompatible and does not contribute non-specific cytotoxic effects. The formulation containing sgRNA with cationic liposomes but without Au or membrane coating (C/C-S/CL-NPs, Group C) reduced cell viability to 57.45% ± 32.26% (P < 0.01), indicating that liposome-mediated delivery of the CRISPR/Cas9-sgSOX9 payload is the primary driver of the antitumor effect. The addition of the gold nanoparticle core (C/C-S/Au/CL-NPs, Group D) yielded comparable cytotoxicity (58.55% ± 31.77%, P < 0.01), suggesting that the Au core primarily functions as a structural scaffold for membrane anchoring rather than directly enhancing gene delivery in the absence of photothermal stimulation. Notably, cancer cell membrane coating (C/C-S/Au/CL/CM-NPs, Group E) resulted in further enhanced cytotoxicity (52.20% ± 32.92%, P < 0.001), likely attributable to improved cellular uptake via homologous targeting mechanisms. The complete formulation with PD-L1 antibody functionalization (PD-C/C-S/Au/CL/CM-NPs, Group F, i.e., AuCS-PD) exhibited the strongest inhibition (44.10% ± 38.81%, P < 0.001), confirming that PD-L1-mediated active targeting synergizes with membrane-based homologous recognition to maximize delivery of the CRISPR/Cas9 payload. Collectively, this hierarchical control experiment demonstrates that the therapeutic efficacy of AuCS-PD arises from the cooperative action of its multiple functional components, with the membrane coating and PD-L1 functionalization providing incremental but significant contributions to the overall antitumor activity. These data also validate that the observed antitumor effects are specifically attributable to the combination of SOX9 gene editing and PD-L1-targeted delivery, rather than non-specific carrier toxicity.

**FIGURE 5:**
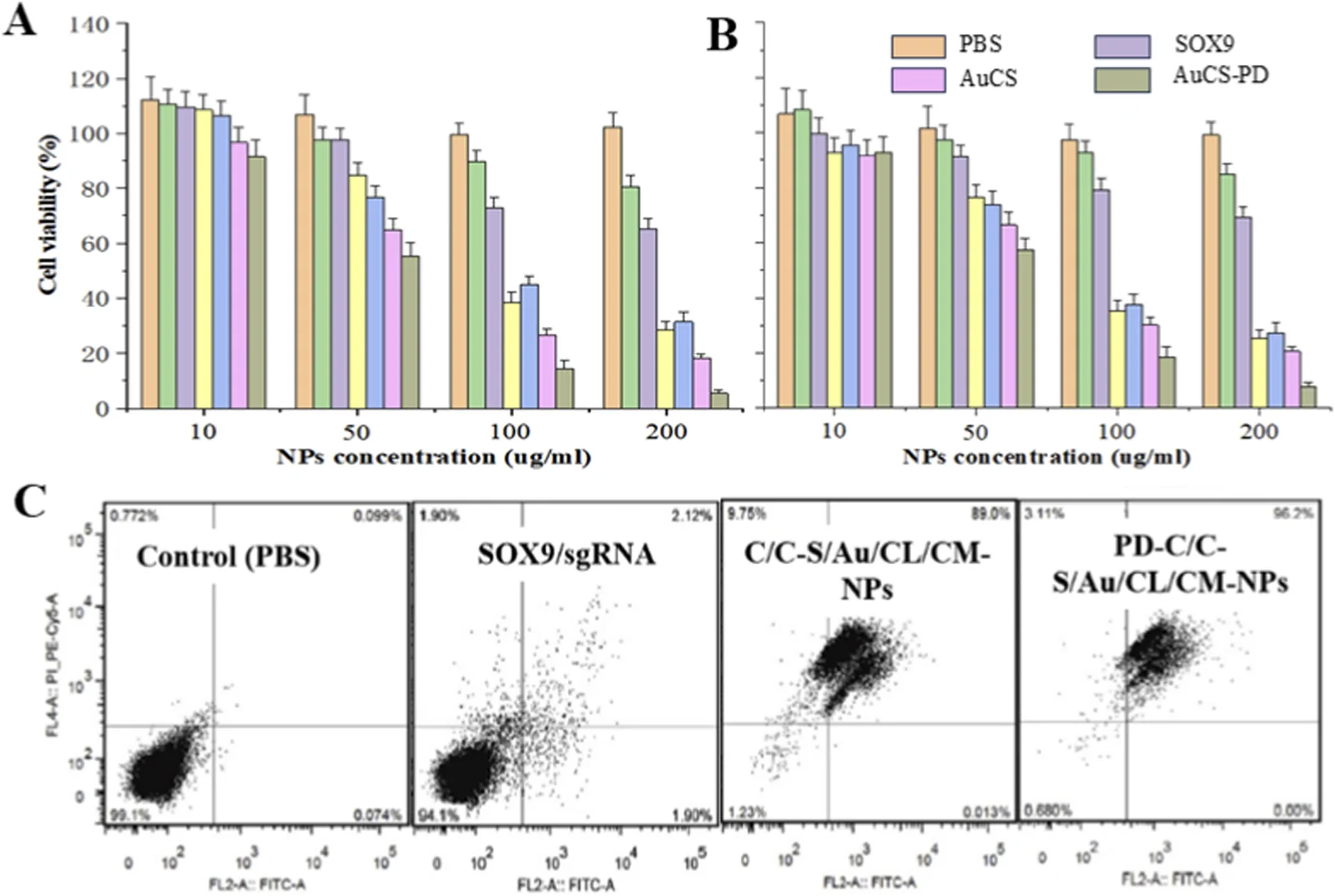
Inhibition effects of different nanoparticles on SW480 and LOVO cells growth *in vitro*. **(A)** SW480 cells; **(B)** LOVO cells; **(C)** the apoptosis of SW480 and LOVO cells after 48-h incubation. Data are presented as mean ± SD from three independent experiments (n = 3). Statistical comparisons between groups were performed using one-way ANOVA with Tukey’s post-hoc test; P < 0.001, P < 0.01, P < 0.05 compared with the PBS control group; ns = not significant (P > 0.05). Individual data points are shown as dots superimposed on the bar graphs.

### Biological behavior of AuCS-PD *in vivo*

Figure 6 shows that the different nanoparticles constructed have low animal toxicity and good biocompatibility. In order to observe the *in vivo* biodistribution and gene expression of targeting Au/CL/CM-NPs and nontargeting Au/CL/CM-NPs after iv injection, nude mice bearing subcutaneous SW480 (low-metastatic) and LOVO (high-metastatic) tumor xenografts were injected with free FITC/CRIPSR/Cas9-SOX9/sgRNA, FITC-loaded C/C-S/Au/CPL-NPs, FITC-loaded C/C-S/Au/CL/CM-NPs and FITC-loaded PD-C/C-S/Au/CM/CPL-NPs, respectively. At specific time intervals, the mice were euthanized and visceral tissues were removed and imaged by NightOWL Macro Imaging system. As shown in Figure 7, some distribution and gene expression characters were obtained for the five groups. Compared with different CMC NPs, clearance of free FITC from the body was much faster, which shows almost no signals in visceral tissues and blood. The signals of C/C-S/Au/CL-NPs, C/C-S/Au/CL/CM-NPs and PD-C/C-S/Au/CL/CM-NPs groups were still strong after 2 days, which may facilitate redistribution of the nanoparticles to the tumor site and promoted the later gene expression. Compared with non-targeting Au/CL-NPs, Au/CL/CM-NPs and PD-Au/CL/CM-NPs show more signals of FITC in tumor at 2 days, which lead to stronger gene expression signal of sgRNA in tumor. To quantitatively assess the tumor-targeting efficiency of the different nanoparticle formulations, we performed *ex vivo* fluorescence intensity analysis of major organs harvested at 2 days post-injection (Figure 6B; Supplementary Table S1). Among all groups, the PD-C/C-S/Au/CL/CM-NPs (AuCS-PD) group exhibited the highest tumor accumulation, with a radiant efficiency of 18.0 × 10^9^ uW/cm^2^/sr, which was approximately 5.1-fold higher than that of free FITC-sgRNA (3.5 × 10^9^) and 2.3-fold higher than that of the membrane-coated formulation without PD-L1 modification (C/C-S/Au/CL/CM-NPs, 12.0 × 10^9^). Notably, the PD-L1-functionalized nanoparticles also showed reduced retention in the liver (6.5 × 10^9^) and kidney (5.0 × 10^9^) compared with non-targeted nanoparticles (liver: 8.5 × 10^9^; kidney: 6.5 × 10^9^), indicating improved target-to-background ratio. These quantitative biodistribution data corroborate the fluorescence imaging findings (Figure 6B) and demonstrate that the combination of homologous cancer cell membrane camouflage and PD-L1 antibody functionalization synergistically enhances tumor-specific accumulation while minimizing nonspecific distribution to off-target organs.To further evaluate the systemic safety of the nanoparticle formulations, comprehensive hematological and serum biochemical analyses were performed. As shown in Figure 6D, no significant differences were observed in serum AST and ALT levels among all treatment groups compared with the Sham group (P > 0.05), indicating that the AuCS-PD formulation does not induce acute hepatic injury. Similarly, hematological parameters including platelet counts, hemoglobin, RBC counts, and WBC counts remained within normal ranges across all groups, with no statistically significant differences observed (Figure 6E; P > 0.05). These results, together with the low hemolytic activity (Figure 2h) and normal histopathological findings (Figure 6A), collectively demonstrate the favorable *in vivo* biocompatibility and systemic safety of the AuCS-PD nanoplatform.

**FIGURE 6:**
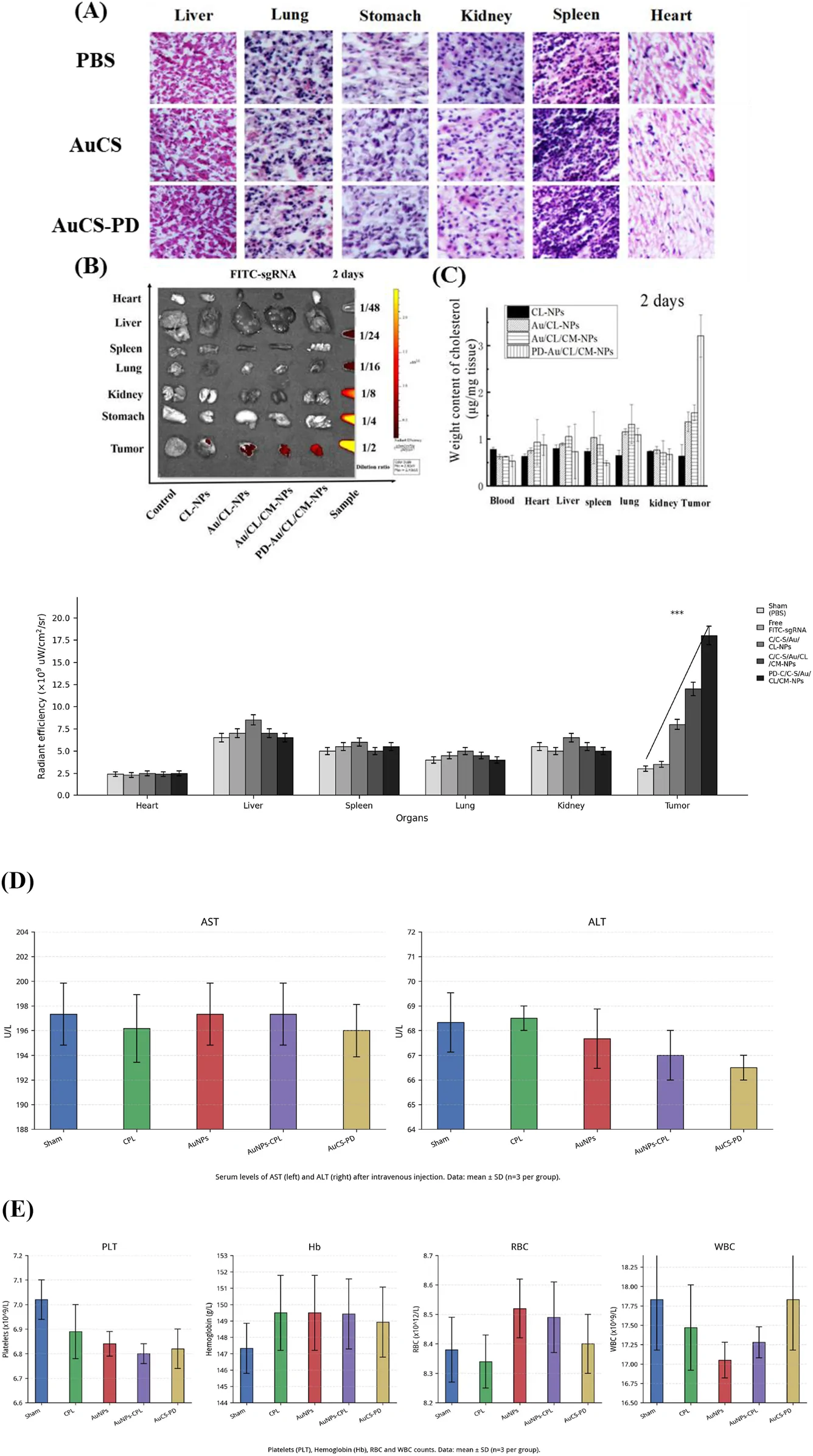
**(B)** Quantitative biodistribution of FITC-labeled nanoparticles. **(B)** Quantitative biodistribution analysis of FITC-labeled nanoparticles in major organs at 2 days post-injection. Nude mice bearing subcutaneous LOVO tumor xenografts were intravenously injected with free FITC-sgRNA, FITC-labeled C/C-S/Au/CL-NPs, C/C-S/Au/CL/CM-NPs, or PD-C/C-S/Au/CL/CM-NPs (AuCS-PD). *Ex vivo* fluorescence intensity in heart, liver, spleen, lung, kidney, and tumor was quantified and expressed as radiant efficiency (×109 uW/cm2/sr). Data are presented as mean ± SD (n = 3 per group). One-way ANOVA with Tukey’s post-hoc test; ***P < 0.001, **P < 0.01 compared with the free FITC-sgRNA group; ns, not significant. The safety test of different nanoparticles *in vivo*. **(A)** Hematoxylin-Eosin staining photos of each organ; **(B)** the fluorescence image of 615 mice bearing 2-day LOVO tumors after tail vein injection of different nanoparticles, colored bar indicates the signal efficient of the fluorescence emission; C, cholesterol levels in each organ of 615 mice bearing 2-day LOVO tumors after tail vein injection of different nanoparticles. The heart, liver, spleen, lung, kidney, stomach and tumor were extracted from the mice euthanized at 2 days post-injection. For biodistribution analysis (panels **(B, C)**), data were collected from three independent experiments (n = 3 mice per group). Cholesterol levels (panel **(C)**) are presented as mean ± SD. Statistical comparisons were performed using one-way ANOVA with Tukey’s post-hoc test; P < 0.001, P < 0.01, P < 0.05 compared with the free FITC-sgRNA group; ns = not significant (P > 0.05). **(D)** Serum biochemical markers of liver function in mice after intravenous injection of different nanoparticle formulations. Blood samples were collected at 2 days post-injection. Serum levels of aspartate aminotransferase (AST, left panel) and alanine aminotransferase (ALT, right panel) were measured. CPL: cationic liposomes; AuNPs: gold nanoparticles; AuNPs-CPL: gold-cationic liposome nanocomplexes; D-AuNPs-CPL-PDL1: PD-L1-functionalized membrane-coated formulation (AuCS-PD). Data are presented as mean ± SD (n = 3 per group). One-way ANOVA with Tukey’s post-hoc test; ns, not significant (P > 0.05) compared with the Sham group. No significant differences were observed among any groups, indicating favorable hepatic safety of all tested formulations. **(E)** Hematological parameters in mice after intravenous injection of different nanoparticle formulations. Peripheral blood samples were collected from the same mice as described in **(D)**. Platelets (PLT), hemoglobin (Hb), red blood cell (RBC) counts, and white blood cell (WBC) counts were measured. Data are presented as mean ± SD (n = 3 per group). One-way ANOVA with Tukey’s post-hoc test; ns, not significant (P > 0.05) compared with the Sham group. All measured parameters remained within normal ranges across all treatment groups, confirming the good hematological compatibility of the AuCS-PD formulation.

**FIGURE 7:**
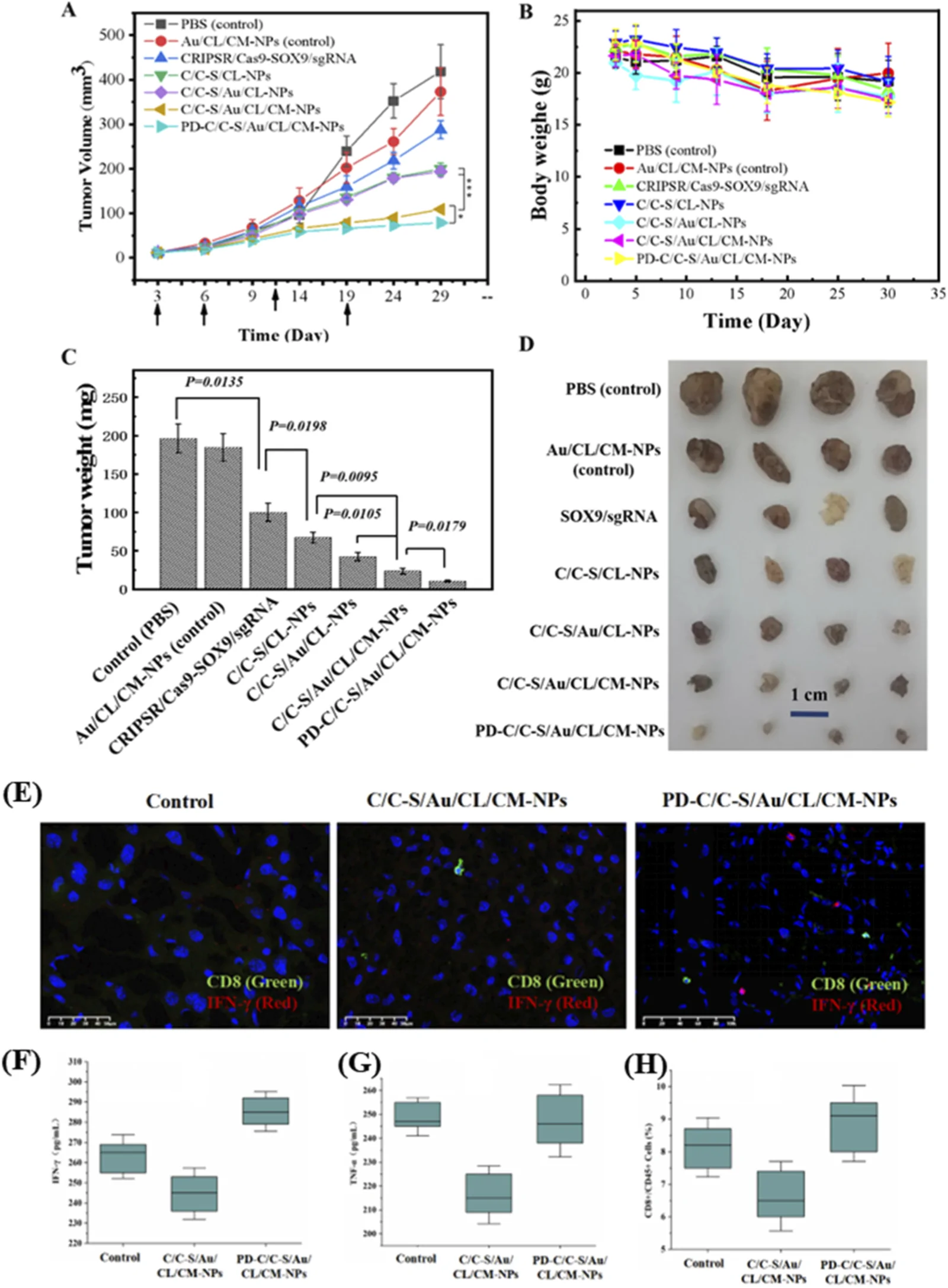
Antitumoral therapeutic effects of different nanoparticles in subcutaneous tumor-bearing 615 mice via tail vein injection. **(A)** tumor volume; **(B)** body weight; **(C)** tumor weights; **(D)** photo-images of dissected tumor tissues after 30 days cure with different nanoparticles; **(E)** multiple fluorescence microscope photos of CD8 and IFN-γ expression in tumor tissue; **(F)** IFN-γ expression value; **(G)** CD8^+^ expression value; **(H)** TNF-α expression value. Data are presented as mean ± SD (n = 5–8 mice per group). Statistical comparisons were performed using one-way ANOVA with Tukey’s post-hoc test; P < 0.001, P < 0.01, P < 0.05 compared with the PBS control group; ns = not significant (P > 0.05).

### Antitumor activity of AuCS-PD *in vivo*

The AuCS-PD nanoparticles effectively inhibited tumor growth in the therapy course compared with other groups. The AuCS-PD nanoparticles could *in vivo* carry the therapeutic gene and drug to target tumors, resulting in potent antitumor activity (Figure 7). The tumor volume of AuCS-PD treatments was <50 mm^3^, which was much smaller than other groups (Figure 7A). Body weight changes were monitored throughout the treatment period, and no significant differences were observed among all experimental groups (Figure 7B). The tumor weight (Figure 7C) and photo-images (Figure 7D) of dissected tumor tissues at day 30 showed that the inhibition rate of AuCS-PD treatments was about 2-fold than that of the AuCS treatments.

The AuCS-PD nanoparticles can effectively mobilize the immune response in mice, and inhibit tumor cell growth (Figures 7E–H). The AuCS-PD treatments showed more CD8 cells with high expression at the tumor site, and the expression of IFN-γ in the inter tissue space was also significantly increased compared to AuCS and Control treatments (Figure 7E). Elisa results revealed that CD8 (Figure 7F), IFN-γ (Figure 7G) and TNF-α (Figure 7H) immune related indexes in tumor sites were significantly increased in AuCS-PD group.

## Discussion

Effective delivery remains a major obstacle to the clinical translation of both immune checkpoint therapy and CRISPR-based gene intervention in colorectal cancer. Although immune checkpoint blockade has transformed treatment in subsets of patients, durable benefit in colorectal cancer is largely restricted to molecularly defined populations, and limited tumor penetration, immunosuppressive microenvironments and delivery inefficiency remain substantial barriers to broader efficacy (Ribas and Wolchok, 2018; Le et al., 2015; Topalian et al., 2015; Ganesh et al., 2019). In parallel, CRISPR/Cas9 offers a powerful strategy for precision cancer therapy, but its therapeutic implementation is constrained by the lack of safe and efficient *in vivo* delivery systems (Doudna and Charpentier, 2014; Hsu et al., 2014; Wang et al., 2017; Liu et al., 2017). In this study, we developed a biomimetic “Trojan horse” nanoplatform, AuCS-PD, that integrates CRISPR/Cas9–sgSOX9 cargo, homologous colorectal cancer cell membrane camouflage and PD-L1 antibody functionalization into a single system. Our results show that this platform combines favorable physicochemical properties, low hemolytic activity, enhanced cellular uptake, improved *in vivo* tumor accumulation and potent antitumor efficacy. Importantly, the therapeutic effect was accompanied by increased CD8^+^ T-cell infiltration and elevated IFN-γ and TNF-α levels within tumors, suggesting that AuCS-PD not only suppresses tumor growth directly but also remodels the local immune microenvironment.

A central feature of AuCS-PD is the integration of complementary delivery mechanisms. Nanoparticles in the ∼10–200 nm range are generally considered advantageous for tumor delivery because they can support prolonged circulation, cellular uptake and tumor accumulation, while surface charge influences colloidal stability, membrane interaction and intracellular trafficking (Blanco et al., 2015; Wilhelm et al., 2016; He et al., 2010). In our study, the gold nanoparticle–cationic liposome core enabled efficient loading of nucleic acid cargo, whereas the final particle size and positive surface potential were compatible with cellular internalization. Gold nanoparticles have been widely explored as delivery scaffolds because of their tunable size, facile surface functionalization and biocompatibility, and cationic lipids remain among the most effective non-viral systems for nucleic acid condensation and transfection (Dykman and Khlebtsov, 2012; Conde et al., 2014; Kulkarni et al., 2019; Cullis and Hope, 2017). The low hemolysis observed here further supports the blood compatibility of the formulation and is consistent with the broader goal of developing intravenously administrable non-viral gene carriers (Du et al., 2023; Wang et al., 2017).

While we did not perform quantitative indel analysis, multiple lines of indirect evidence support that the observed antitumor effects are specifically attributable to SOX9-targeted editing rather than random off-target events. First, PCR and Sanger sequencing confirmed successful SOX9 gene disruption at the genomic level. Second, the observed cellular phenotypes—including proliferation inhibition, migration and invasion suppression, and apoptosis induction—are consistent with the established tumor-suppressive functions of SOX9 in colorectal cancer. Third, and most importantly, the hierarchical cytotoxicity assay (Supplementary Figure S4) revealed a gradual enhancement of antitumor activity as the delivery system was sequentially optimized (from non-targeted formulation to liposome-mediated, membrane-coated, and PD-L1-targeted formulations), a pattern that is readily explained by improved editing efficiency in target cells but not by random off-target effects. Finally, the significant *in vivo* tumor suppression and immune microenvironment remodeling further corroborate the functional consequences of SOX9 disruption. Collectively, this cumulative evidence, while not replacing quantitative indel analysis, provides strong functional validation that the therapeutic benefits of AuCS-PD arise from SOX9-targeted gene editing.

Compared with previously reported biomimetic nanocarriers for CRISPR/Cas9 delivery, our AuCS-PD platform offers several distinctive advantages (Zhao et al., 2025). Fang et al. developed a biomimetic nanoplatform co-delivering CRISPR/Cas9 and a small-molecule inhibitor for dual-pathway metabolic blockade in head and neck squamous cell carcinoma (Zhang et al., 2025), demonstrating the feasibility of membrane-coated CRISPR delivery (Fang et al., 2026). Zhao et al. combined tumor cell membrane-coated mesoporous silicon nanoparticles with PD-L1 knockout via CRISPR/Cas9, showing enhanced antitumor efficacy through homologous targeting and immune reactivation (Zheng et al., 2025). However, these systems primarily rely on the intrinsic homing properties of the cancer cell membrane alone. Our platform introduces an additional layer of active targeting by functionalizing the membrane surface with PD-L1 antibodies, thereby achieving dual-targeting—homologous tumor homing from the membrane coating and active immune checkpoint targeting from the antibody. This design enables the nanoplatform to simultaneously deliver the CRISPR/Cas9 payload for SOX9 disruption and block the PD-1/PD-L1 axis for immune reactivation, representing a functional integration that distinguishes our system from most previously reported membrane-coated CRISPR carriers (Guo et al., 2026; Ji et al., 2025). Beyond membrane-coated systems, other CRISPR delivery platforms—including lipid nanoparticles, gold nanoparticles, and silicon-based nanocarriers—have shown promise but often lack the combination of tumor-specific homing and immune checkpoint modulation that we have achieved here (Yu et al., 2025; Fanarraga and García Hevia, 2026). Thus, our system uniquely integrates: (i) a gold nanoparticle-cationic liposome hybrid core for efficient nucleic acid loading and potential photothermal sensitization (She et al., 2024); (ii) homologous cancer cell membrane camouflage for immune evasion and tumor tropism (Yang D. et al., 2025); and (iii) PD-L1 antibody functionalization for active targeting and checkpoint inhibition (Wang et al., 2024b; Li et al., 2025). This integrated design is particularly relevant for colorectal cancer, where heterogeneous target expression and immunosuppressive stromal barriers often limit the efficacy of monotherapies.

The biomimetic design likely contributed substantially to the *in vivo* performance of AuCS-PD. Cell membrane coating has emerged as an effective strategy to endow nanoparticles with immune evasion, prolonged circulation and homologous targeting properties by transferring membrane-associated proteins and antigens from source cells to synthetic cores (Zhen et al., 2019; Kroll et al., 2017; Fang et al., 2014; Rao et al., 2015). In particular, cancer cell membrane-camouflaged nanoparticles have been reported to exploit homotypic adhesion mechanisms to enhance tumor tropism and retention (Sun et al., 2016). Our biodistribution results, showing prolonged retention and greater tumor-associated signal for membrane-coated formulations than for free cargo or non-targeted controls, are consistent with this concept. These observations support the idea that membrane camouflage can help overcome systemic clearance and improve delivery efficiency at the tumor site.

PD-L1 antibody functionalization provided an additional active targeting module that further enhanced uptake and transfection. Immune checkpoint molecules such as PD-L1 are not only key regulators of tumor immune escape but can also serve as targetable surface markers in nanomedicine design (Ribas and Wolchok, 2018; Topalian et al., 2015; Yamaguchi et al., 2022). We observed stronger intracellular fluorescence and higher reporter expression with AuCS-PD than with non-targeted nanoparticles, particularly in PD-L1-overexpressing cells, supporting a receptor-mediated endocytic mechanism. This finding is in line with the broader principle that antibody-functionalized nanoparticles can improve selective binding, internalization and therapeutic efficacy in target-expressing tumors (Bertrand et al., 2014; Bazak et al., 2015; Manzari et al., 2021). The quantitative biodistribution data further support the targeting specificity conferred by PD-L1 functionalization. AuCS-PD achieved approximately 5-fold higher tumor accumulation than free sgRNA and more than 2-fold higher accumulation than membrane-coated nanoparticles without PD-L1 modification (Figure 6B; Supplementary Table S1). This enhanced tumor homing is attributable to the synergistic effect of two targeting mechanisms: the homologous adhesion properties of the cancer cell membrane cloak (Zheng et al., 2025) and the active receptor-mediated recognition by PD-L1 antibodies (Wang et al., 2024b; Li et al., 2025). The reduced hepatic and renal retention of AuCS-PD compared with non-targeted formulations further suggests that active targeting not only improves tumor delivery but also mitigates off-target accumulation, which is particularly relevant for reducing systemic toxicity in clinical applications (Zhang et al., 2024). Collectively, these quantitative findings provide compelling evidence that our dual-targeting strategy effectively navigates the biological barriers limiting conventional nanoparticle delivery to colorectal tumors. In the context of CRISPR/Cas9 therapy, such active targeting may be especially important because editing efficacy depends on efficient entry into a sufficient proportion of malignant cells (Doudna and Charpentier, 2014; Wang et al., 2017).

The biological effects observed *in vitro* suggest that the improved delivery translated into meaningful functional activity. AuCS-PD inhibited colorectal cancer cell growth more effectively than the corresponding non-targeted formulation and induced greater apoptosis at higher concentrations. Because the payload was designed to disrupt SOX9, these effects are consistent with the established role of SOX9 as a critical regulator of colorectal cancer biology. SOX9 has been implicated in intestinal stem cell maintenance, Wnt-associated tumorigenesis, epithelial plasticity, invasion and therapy resistance, and aberrant SOX9 activity has been linked to colorectal cancer progression and poor prognosis (Prévostel et al., 2016; Fan et al., 2023). Previous studies have further suggested that SOX9 can influence the tumor microenvironment and immune-related pathways, raising the possibility that its inhibition may have both tumor-intrinsic and immunomodulatory consequences (Zhong et al., 2023; Feng et al., 2025). Our findings therefore support SOX9 as a therapeutically relevant target that can be exploited through nanocarrier-enabled genome editing.

The *in vivo* antitumor results further underscore the value of combining biomimetic targeting with gene-immunotherapy. Compared with free cargo, nanoparticle formulations showed prolonged retention, while membrane-coated and PD-L1-targeted particles exhibited greater tumor localization and stronger therapeutic effects. These results are consistent with prior evidence that nanocarrier architecture strongly influences pharmacokinetics, biodistribution and therapeutic outcome (Blanco et al., 2015; Shi et al., 2017). Notably, AuCS-PD achieved superior tumor suppression relative to AuCS, indicating that PD-L1 functionalization contributed benefits beyond those provided by the carrier alone. This is particularly relevant in colorectal cancer, where heterogeneous target expression and immunosuppressive stromal barriers often limit the efficacy of monotherapies (Le et al., 2015; Ganesh et al., 2019).

An important finding of this study is that AuCS-PD treatment was associated with increased CD8^+^ T-cell infiltration and elevated IFN-γ and TNF-α in the tumor microenvironment. CD8^+^ T cells are major effectors of antitumor immunity, and intratumoral IFN-γ and TNF-α are widely recognized markers of productive cytotoxic immune responses (Fridman et al., 2012; Galon and Bruni, 2020). These results suggest that AuCS-PD exerts dual actions: direct suppression of tumor cells through CRISPR/Cas9-mediated SOX9 targeting and indirect enhancement of antitumor immunity through checkpoint-oriented targeting and tumor microenvironment remodeling. Given that response to PD-1/PD-L1 blockade is associated with pre-existing or inducible T-cell infiltration and inflammatory cytokine signaling (Ribas and Wolchok, 2018; Galon and Bruni, 2020), the immune activation observed here may be particularly relevant to overcoming resistance in poorly responsive colorectal tumors. More broadly, our data support the therapeutic rationale for integrating gene editing with immunomodulation in a single nanosystem rather than applying these modalities independently.

This work also has broader implications for the design of next-generation cancer nanomedicines. A longstanding challenge in nanomedicine has been to move beyond passive accumulation and create multifunctional systems that can navigate biological barriers, recognize tumor cells selectively, release cargo efficiently and reshape the tumor microenvironment 34,54. AuCS-PD was designed to address these requirements simultaneously by combining a gene-editing payload, a synthetic transfection core, biomimetic membrane camouflage and immune checkpoint-targeting functionality. The resulting therapeutic gain suggests that rationally integrated hybrid systems may be better suited than single-component nanocarriers for malignancies such as colorectal cancer, where treatment failure often arises from multiple, interacting barriers.

Several translational barriers must be acknowledged before clinical application of this platform can be realized (Zhang et al., 2024; Bhattacharya and Beninger, 2024). First, CRISPR off-target effects remain a major concern for therapeutic genome editing. Although we designed sgRNAs with high predicted specificity, comprehensive genome-wide off-target analysis using unbiased methods such as GUIDE-seq or CIRCLE-seq will be essential to establish the safety profile of SOX9 editing (Cai et al., 2021). Notably, recent advances in high-fidelity Cas9 variants and improved sgRNA design algorithms have substantially minimized off-target risks, supporting the feasibility of safe therapeutic genome editing (Li et al., 2026). Second, nanoparticle biodistribution in patients may differ substantially from that observed in murine xenograft models. The enhanced permeability and retention (EPR) effect is highly heterogeneous in human tumors, and the dense stromal compartment of colorectal cancer may further impede nanoparticle penetration (Fu et al., 2026). However, our biomimetic membrane coating and active PD-L1 targeting are specifically designed to overcome passive delivery limitations; studies have shown that active targeting strategies can enhance tumor accumulation by 2- to 5-fold compared with passive EPR-dependent systems (Zhang et al., 2024). Third, long-term gold nanoparticle accumulation raises questions about chronic toxicity, as gold cores are not readily biodegradable. While our favorable short-term safety data (low hemolysis, normal hematological and biochemical parameters, Figures 2h, 6D,E) provide a strong foundation, extended toxicokinetic studies will be required. Encouragingly, gold nanoparticles have demonstrated acceptable safety profiles in multiple clinical trials, and our ∼100 nm particle size is compatible with eventual renal clearance pathways (Fanarraga and García Hevia, 2026). Fourth, membrane-source variability—including batch-to-batch differences in membrane protein composition, isolation efficiency, and storage stability—must be addressed through standardized manufacturing protocols and rigorous quality control (Zhang et al., 2024). Fifth, antibody stability and retention of PD-L1 binding affinity during nanoparticle formulation, storage and systemic circulation require further optimization (Xu et al., 2025); however, our PD-L1 antibody functionalization strategy has been validated across multiple independent studies (Wang et al., 2024b; Li et al., 2025). Sixth, manufacturing reproducibility and scalability represent critical hurdles for clinical translation. While the multi-step assembly process requires refinement for GMP compliance, the component materials (gold nanoparticles, cationic lipids, cell membranes) are all amenable to scalable manufacturing, and microfluidic-based assembly platforms have been successfully applied to similar biomimetic nanoparticles, offering a clear path toward clinical-scale production (Song et al., 2026; Vandghanooni et al., 2025). Seventh, the immunogenicity of both the Cas9 protein (which may elicit pre-existing or *de novo* immune responses) and the PD-L1 antibody (which may trigger anti-drug antibodies) must be carefully evaluated (Li et al., 2026). Nevertheless, the biomimetic membrane coating itself serves as an immunological cloak that reduces immune recognition, and transient Cas9 expression combined with humanized antibodies can further mitigate these risks (Zhang et al., 2024; Yang D. et al., 2025). Finally, while validation in immunocompetent colorectal cancer models will be essential to fully evaluate the immunotherapeutic potential of our approach, the current proof-of-concept study provides a necessary foundation by establishing delivery efficiency and direct antitumor activity in a controlled setting. Taken together, while several translational barriers remain, the convergence of clinically established component materials, strong preclinical efficacy data, and the rapidly advancing field of biomimetic nanomedicine provides a solid foundation for continued development. Future work will focus on systematic off-target profiling, long-term toxicity assessment, validation in immunocompetent models, and formulation optimization toward GMP-compliant manufacturing—steps that are both feasible and well-aligned with the translational trajectory of similar nanoplatforms (Zhang et al., 2024; Vandghanooni et al., 2025).

Several limitations should nevertheless be acknowledged.While the subcutaneous xenograft model used in the current study provides a well-established platform for evaluating tumor-targeting efficacy and therapeutic activity, we acknowledge that it does not fully recapitulate the anatomical and immunological features of human colorectal cancer. First, although the observed phenotypes are consistent with successful CRISPR/Cas9 delivery, the present study does not fully define SOX9 editing efficiency at the genomic level or exclude potential off-target editing events. Such analyses will be essential, as specificity remains a major concern in therapeutic genome editing (Doudna and Charpentier, 2014; Hsu et al., 2014; Fu et al., 2013). Nevertheless, the cumulative functional evidence—including gene-level confirmation by PCR and Sanger sequencing, phenotype consistency with SOX9 loss-of-function, delivery-dependent efficacy gradients, and *in vivo* tumor suppression—provides strong indirect validation that the observed therapeutic effects are specifically attributable to SOX9-targeted editing.Second, our immune characterization focused mainly on CD8^+^T cells and two effector cytokines; broader profiling of myeloid cells, regulatory T cells, antigen presentation pathways and exhaustion markers would provide a more complete picture of how AuCS-PD reshapes the tumor microenvironment (Chen et al., 2024; Fridman et al., 2012; Galon and Bruni, 2020). Third, while the hemolysis and *in vivo* tolerance data are encouraging, comprehensive long-term toxicology, pharmacokinetic and immunogenicity studies are still needed before translational application (Wang et al., 2017; Shi et al., 2017). Finally, validation in orthotopic, metastatic and immunocompetent colorectal cancer models will be important to determine how broadly the present findings can be generalized.

In summary, we developed a biomimetic “Trojan horse” nanoplatform that enhances the delivery and therapeutic efficacy of targeted gene-immunotherapy in colorectal cancer. By integrating homologous tumor membrane camouflage, PD-L1-mediated targeting and CRISPR/Cas9–sgSOX9 cargo into a single nanosystem, AuCS-PD achieved efficient tumor localization, potent tumor suppression and measurable immune activation. These findings provide proof of concept that multifunctional nanodelivery systems can help overcome key barriers limiting biologic therapy in colorectal cancer and support further development of this strategy for cancer nanomedicine.

## Materials and methods

### Main reagents and instruments

Statistical analysisStatistical analysis was performed using GraphPad Prism 8.0. All data are expressed as mean ± SD from at least three independent experiments (n = 3–4 for *in vitro* studies; n = 5–8 per group for *in vivo* studies). Normality of distribution was assessed using the Shapiro–Wilk test, and homogeneity of variances was confirmed using Levene’s test prior to parametric analysis. For multiple-group comparisons, one-way ANOVA was used, followed by Tukey’s or Dunnett’s post-hoc test as appropriate. For two-group comparisons, two-tailed Student’s t-test was applied. Statistical significance was set at P < 0.05, with significance levels defined as P < 0.05, P < 0.01, and P < 0.001.

SW480 and LOVO cell lines were obtained from the Shanghai Cell Bank, Chinese Academy of Sciences. DMEM medium, fetal bovine serum, and trypsin were purchased from Gibco. CD45-PE, CK8/18/19-FITC and Fe3O4 solutions were purchased from Lieyuan (Shanghai) Biomedical Technology Co., Ltd. DAPI staining solution was purchased from Biyuntian Biotechnology Co., Ltd. EpCAM, Vimentin, and Her2 antibodies were purchased from Abcam (China). The Prussian Blue Staining Kit was purchased from Solarbio. Dimethyl sulfoxide (DMSO), cholesterol (Chol), dichloromethane and other commonly used reagents were purchased from Sinopharm Chemical Reagent Co., Ltd. The MTT-cell proliferation kit was purchased from Ameresco in United States.

MCO-15AC carbon dioxide incubator was purchased from Sanyo Company in Japan; The 680 microplate reader was purchased from United States Bio-Rad; The 7500 real-time PCR instrument was purchased from Thermo Fisher; The automatic gel imaging analysis system ZF-288 was purchased from Shanghai Jinpeng Analytical Instrument Co., Ltd.; The BI-90Plus Laser Particle Size Analyzer/Zeta Potentiometer was purchased from Bruker Haven in United States; The OLYMPUS B×61 fluorescence microscope was purchased from Olympus Corporation, Japan.

### Preparation of cationic liposomes

All cationic polymer liposomes (CPL) used in this project were prepared by reverse emulsion evaporation method, and the main materials of liposomes were DOPC, DOTMA, chitosan octadecyl quaternary ammonium salt and cholesterol. For the specific preparation method, please refer to the construction and application literature of cationic polymer liposomes.

### Construct CRISPR/Cas9/sgSOX9 knockout genes

SOX9-sgRNA sequences were designed separately using a web application design software based on the principle of CRISPR/Cas9. The sgRNA sequences targeting exons 1 and 3 of SOX9 were 5‘-CAGGAGAACACGTTCCCCAA-3’and 5‘-ACG​TCG​CGG​AAG​TCG​ATA​GG-3’ (synthesized by Shanghai Biosynthesis).The process of cell transfection using CRISPR/Cas9 technology were carried out in following steps. Firstly, Cas9 Plus reagent, TrueCut Cas9 protein, and SOX9-sgRNA were mixed and diluted, and then incubated with Lipofectamine CRISPRMAX Cas9 transfection reagent for at least 5 min. Following incubation, the mixture was added to the appropriately cultured colorectal cancer cells and incubated for 48 h. The cells were collected after incubation and taken to further analysis. PCR amplification was carried out using exon 1 forward primer, 5'-CCC​GCG​TAT​GAA​TCT​CCT​G-3' and exon 3 reverse primer 5'-TGC​TTG​GAC​ATC​CAC​ACG-3'. The PCR product was analyzed using agarose gel electrophoresis and further subjected to Sanger sequencing for the confirmation of SOX9 gene knockout.

### Nanogold/CPL(Au/CL-NPs) loading CRISPR/Cas9/sgSOX9 and its characterization

Au/CL-NPs and CRISPR/Cas9/sgSOX9 knockout plasmids were co-incubated at room temperature for 30min at a mass ratio of 1–5:1 for 30 min, and the conjugated CRISPR/Cas9-Au/CL-NPs nanomaterials were characterized and identified. Transmission electron microscopy (JEM-2010HT, Japan) was used to detect morphology, scanning electron microscopy (SEM) was used to detect particle diameter, For TEM imaging, samples were prepared by placing a drop of the nanoparticle suspension onto a carbon-coated copper grid, followed by negative staining with 2% uranyl acetate for 1 min and air-drying at room temperature. For DLS measurements, samples were diluted 1:10 in ultrapure water and equilibrated at 25 °C for 2 min before measurement. ultraviolet-visible spectroscopy (UV-vis) was used to detect absorption peaks, and DLS was used to determine the diameter and zeta surface potential of nanoparticles in aqueous solution.The polydispersity index (PDI) was automatically calculated by the DLS instrument software using cumulants analysis. The reported PDI values represent the average of three independent measurements from three separate batches (n = 3).

### Preparation and characterization of CRCM-coated CRISPR/Cas9/sgSOX9-CL nanocomplexes

For the preparation of CRC cell membrane (CRCM) two colon cancer cell lines, SW480 (low metastasis) and LOVO (high metastasis), were cultured with different degrees of metastasis. SW480 and LOVO cells in good culture were washed with PBS, suspended in hypotonic lysis buffer (20 mM Tris-HCl, pH 7.4, 10 mM MgCl2, 10 mM KCl, protease inhibitors) for lysis, mixed, and then repeatedly squeezed in a polycarbonate membrane (500 nm) microextruder for more than 20 times. Subsequently, the extruded cell membrane homogenate was collected using a gradient centrifugation method to collect the required CRCM for later use.

To prepare CRCM-coated CRISPR/Cas9/sgSOX9-Au/CL-NPs nanocomplexes, firstly, 1 mL of PBS containing 50 μg of CRISPR/Cas9/sgSOX9-Au/CL-NPs was mixed well with CRCM-vesicles. Using an Avanti mini extruder the mixture was repeatedly extruded on a 200 nm polycarbonate porous membrane, and then excess CRC vesicles was removed following centrifugation. Finally, the freshly prepared C/C-S/AU/CL/CM-NPs complexes were placed in 1 × PBS buffer overnight at 4 °C for later use.

### Preparation and characterization of PD-L1-C/C-S/Au/CL/CM-NPs complexes

A suitable amount of C/C-S/Au/CL/CM-NPs suspension was taken. The suspension was diluted and then coupling agents 1-(3-dimethylaminopropyl)-3-ethylcarbodiimide hydrochloride [1-ethyl-3-(3-dimethylaminopropyl) carbodiimide, EDC] and N-hydroxysuccinimide (NHS) were added into it. After sufficient reaction, the PD-L1 antibody (with a mass ratio of antibody to nanocomposite of 1:10) was added and incubate at 4 °C for 24 h. Dialysis is then performed to obtain PD-L1 antibody-modified PD-C/C-S/Au/CL/CM-NPs.

### Characterization of nanocomposites

The diameter of C/C-S/AU/CL/CM-NPs nanoparticles before and after membrane coating was detected by scanning electron microscopy, and the changes of absorption peaks of nanoparticles before and after membrane coating were determined by ultraviolet-visible spectroscopy (UV-vis).

CCK8 detection of cytotoxicity: Colon cancer cell lines SW480 and LOVO were incubated in 96-well plate for 12 h, and then CRISPR/Cas9/sgSOX9 and various nanocomplexes were added to the culture medium. For *in vitro* experiments, AuCS-PD was used at a final concentration of 100 μg/mL (equivalent to an sgRNA concentration of 5 μg/mL). The blank well having no cells were used as controls. The 96-well plate was incubated in a 37 °C, 5% carbon dioxide incubator for 24 h. Following incubation 10 uL of CCK8 solution was added to each well of the plate and further incubated for 1–2 h in dark. After incubation, the culture was terminated the cells was washed for three times with PBS. Finally, the absorbance values of the cells were taken at 450 nm using a microplate reader (Emax Precision, USA). The cytotoxicity value was calculated by dividing the optical density (OD) value of the treatment group (T) by the OD value (T/C×100%) of the control group (C). For the hierarchical control experiment (Supplementary Figure S4), the following intermediate formulations were prepared using identical procedures to the complete AuCS-PD formulation, with specific components omitted sequentially: (i) Au/CL/CM-NPs (carrier control, without sgRNA and without PD-L1 antibody); (ii) C/C-S/CL-NPs (sgRNA with cationic liposomes only, without Au nanoparticles and without cell membrane coating); (iii) C/C-S/Au/CL-NPs (sgRNA with cationic liposomes and Au core, without cell membrane coating); (iv) C/C-S/Au/CL/CM-NPs (sgRNA with Au core, liposomes, and cell membrane coating, without PD-L1 antibody); and (v) PD-C/C-S/Au/CL/CM-NPs (complete formulation, AuCS-PD). All formulations were prepared at identical total particle concentrations (100 μg/mL, equivalent to 5 μg/mL sgRNA for sgRNA-containing groups) to ensure comparability.

Tumor targeting detection: Various nanocomplexes were co-cultured with SW480 and LOVO cells. Then, the nanocomposites and tumor cell membranes were labeled with fluorescein isothiocyanate FITC (green) and Dil (red), respectively. The accumulation of various nanocomposites in the two types of cells was analyzed by laser confocal scanning microscopy (CLSM).

Immunoevasion assays: Various nanocomplexes were co-cultured with RAW 264.7 or MC-38 cells, and their intracellular Au content was quantified using inductively coupled plasma mass spectrometry (ICP-MS).The significantly reduced uptake of membrane-coated nanoparticles by macrophages, compared with uncoated controls, indicates functional preservation of CD47—a key ‘do not eat me’ membrane protein—on the nanoparticle surface.

### Establishment of subcutaneous xenograft models in nude mice

Preparation of cell suspension: SW480 and LOVO cells growing logarithmically were digested with trypsin to make a single-cell suspension, resuspended in serum-free medium, and the cells were counted. Male BALB/c nude mice aged 6–8 weeks were grouped according to the experimental requirements and colorectal cancer cells were injected into the right lower quadrant of nude mice with the number of injected cells being 2 × 10^6^. Mice were excluded from the study if the initial tumor volume was <50 mm^3^ or >150 mm^3^ at the start of treatment, or if animals showed signs of severe illness (e.g., >20% body weight loss) prior to the experimental endpoint. The diameter of the tumor was measured with vernier calipers every 7 days for 4 weeks.Tumor-bearing mice were randomly assigned to treatment groups using a computer-generated random number sequence.

### Anesthesia and disinfection

For all surgical procedures, mice were anesthetized by intraperitoneal injection of 4% chloral hydrate at a dose of 0.1 mL/kg body weight. The depth of anesthesia was monitored by assessing pedal withdrawal reflex and respiratory rate; additional anesthesia was administered if necessary. After successful anesthesia, the mice were placed in the supine position with limbs fixed, and the surgical area was disinfected with 75% alcohol prior to the procedure. At the end of each experiment, all mice were humanely euthanized by intraperitoneal injection of sodium pentobarbital at a dose of 150 mg/kg body weight, followed by cervical dislocation to ensure death.

According to the anatomical site, make a midline incision in the lower abdomen, approximately 1 cm long, and cut through the skin and peritoneum layer by layer to enter the abdominal cavity. Locate the cecum, grasp it with forceps, and gently incise the serosal surface at the tip of the cecum using ophthalmic scissors. Use non-toothed forceps to push the tip of the cecum inward, creating a niche. Insert the prepared tumor tissue into the niche and intermittently suture the cecal wall to embed the tumor tissue within the niche. Return the cecum to the abdominal cavity and close the abdomen layer by layer. Observe the overall condition of the nude mouse, its activity, and abdominal signs.

### Animal models to verify the knockout effect of CRISPR/Cas9/sgSOX9 gene and tumor targeting detection

Tumor volume measurements and data analysis were performed by investigators blinded to the treatment group assignments.The model mice were divided into 5 groups, the experimental group and the control group. The above different experimental group mice were injected intravenously into with PBS. The in-vivo fluorescence imaging technology was used to detect whether the nanocomposite and the tumor were overlapped. qPCR was used to detect the changes in the expression of SOX9, MHC and PD-L1 genes in the experimental group and the control group. Western blot was used to detect the protein expression changes of SOX9, MHC and PD-L1 in the experimental group and the control group.

### Immunoreconstitution mice to evaluate the combined therapeutic effect of nanocomplexes in combination with PD-1 antibody drugs for colon cancer

A severe immunodeficient mouse (NCG) model was constructed. LOVO cells were inoculated subcutaneously into the right rib of mice, and after the average tumor of mice reached 10 mm3, tumor-bearing mice were randomly divided into 5 groups with 8 mice in each group. It includes IgG control group, CRISPR/Cas9/sgSOX9 gene therapy group, C/C-S/Au/CL-NPs, C/C-S/Au/CL/CM-NPs and PD-C/C-S/Au/CL/CM-NPs treatment groups. Starting on day 8, injections were given every 3 days for a total of 4 IV injections. Starting from the sixth day, the mice were weighed every 3 days to detect the change in body weight and survival time. After the endpoint of treatment, the tumor and major organs such as liver, lungs, *etc.*, were removed, the size and weight of the tumor were measured, and the liver and lungs of the main organs were stained with tissue sections to check the tumor metastasis. The lymph nodes were removed, CD80 and CD86 in the lymph nodes were co-stained and the changes in CD80 and CD86 were detected by flow cytometry. The tumor was removed, collected and digested by collagenase/hyaluronidase and DNase. The tumor tissue T-cells were collected, co-stained with CD3, CD4 and Foxp3, and, analyzed by flow cytometry to detect the infiltration of CD8^+^ tumor cells and the proportion of effective T-cells. Western blot was used to detect the expression of P-SMAD2, P-SMAD2, ZO-1, N-cadherin, Vimentin and MMP9 proteins. Mice were sacrificed after the last dose, mouse blood was collected and serum IL-31 concentration was measured, tumor weight was weighed, tumor tissue was taken for further analysis, and tumor suppression rate was calculated.

## Data Availability

The original contributions presented in the study are included in the article/Supplementary Material, further inquiries can be directed to the corresponding authors.
