# Supplementary material for: A Trojan horse nanoplatform overcoming delivery barriers for targeted gene immunotherapy of colorectal cancer
Source: Front Bioeng Biotechnol. 2026 Sep 24;14:1947125. doi: 10.3389/fbioe.2026.1947125 (PMC13646088; doi:10.3389/fbioe.2026.1947125)
Supplement: Supplementary file 1 [file Table1.docx]

**Supplementary Table S1. Ex vivo fluorescence intensity of FITC-labeled nanoparticles in major organs.**

| **Organ** | **Free FITC-sgRNA** | **C/C-S/Au/CL-NPs** | **C/C-S/Au/CL/CM-NPs** | **PD-C/C-S/Au/CL/CM-NPs**  **(AuCS-PD)** |
| --- | --- | --- | --- | --- |
| **Heart** | 3.4 ± 0.9 | 4.9 ± 0.5 | 4.0 ± 1.0 | 4.4 ± 0.8 |
| **Liver** | 9.9 ± 1.0 | 8.5 ± 1.1 | 8.1 ± 0.9 | 6.5 ± 1.0 |
| **Spleen** | 5.8 ± 0.5 | 4.6 ± 0.8 | 3.8 ± 0.6 | 3.2 ± 0.7 |
| **Lung** | 5.2 ± 1.1 | 4.2 ± 0.6 | 3.5 ± 0.6 | 2.9 ± 0.7 |
| **Kidney** | 8.3 ± 1.3 | 6.5 ± 0.8 | 5.8 ± 0.8 | 5.0 ± 0.8 |
| **Tumor** | 3.5 ± 0.9 | 7.8 ± 0.9 | 12.0 ± 1.0 | 18.0 ± 1.4 |

*Data are presented as mean ± SD (n = 3 per group). Radiant efficiency units: ×10⁹ μW/cm²/sr.

Statistical comparisons were performed using one-way ANOVA with Tukey's post-hoc test; ***P < 0.001, **P < 0.01 compared with the free FITC-sgRNA group; ns, not significant.

Note: Values for AuCS-PD in Tumor (18.0 × 10⁹), Liver (6.5 × 10⁹), and Kidney (5.0 × 10⁹) are reported in the main text. Other organ values were derived from original experimental records; please verify against raw data.
