# Supplementary material for: A Trojan horse nanoplatform overcoming delivery barriers for targeted gene immunotherapy of colorectal cancer
Source: Front Bioeng Biotechnol. 2026 Sep 24;14:1947125. doi: 10.3389/fbioe.2026.1947125 (PMC13646088; doi:10.3389/fbioe.2026.1947125)
Supplement: Supplementary file 2 [file Table2.docx]

**Supplementary Table S2. Summary of nanoparticle formulations and their components**

| **Formulation** | **Abbreviation** | **sgRNA** | **Au NPs** | **Cationic liposomes** | **CRC cell membrane** | **PD-L1 antibody** | **Experimental purpose** |
| --- | --- | --- | --- | --- | --- | --- | --- |
| Phosphate-buffered saline | PBS | - | - | - | - | - | Untreated control |
| Gold-cationic liposome-CRC membrane nanoparticles | Au/CL/CM-NPs | - | + | + | + | - | Carrier control (without sgRNA and PD-L1) |
| CRISPR/Cas9-sgRNA with cationic liposomes | C/C-S/CL-NPs | + | - | + | - | - | sgRNA delivery baseline (without Au and membrane) |
| CRISPR/Cas9-sgRNA with Au and cationic liposomes | C/C-S/Au/CL-NPs | + | + | + | - | - | Assess Au core contribution (without membrane) |
| CRISPR/Cas9-sgRNA with Au, liposomes, and CRC membrane | C/C-S/Au/CL/CM-NPs | + | + | + | + | - | Assess membrane coating contribution (without PD-L1) |
| PD-L1-functionalized CRISPR/Cas9-sgRNA with Au, liposomes, and CRC membrane | PD-C/C-S/Au/CL/CM-NPs (AuCS-PD) | + | + | + | + | + | Complete formulation |

*The symbols + and – indicate the presence or absence of each component, respectively. CRC, colorectal cancer; CPL, cationic polymer liposomes; CRCM, CRC cell membrane. AuCS-PD (PD-C/C-S/Au/CL/CM-NPs) represents the complete formulation used for all in vivo therapeutic experiments. This table corresponds to the hierarchical control experiment presented in Supplementary Fig. S3. All formulations were prepared at identical total particle concentrations (100 μg/mL, equivalent to 5 μg/mL sgRNA for sgRNA-containing groups).*
